# South Sudan's road to universal health coverage: a slow but steady journey

**DOI:** 10.11604/pamj.supp.2022.42.1.34035

**Published:** 2022-06-07

**Authors:** Victoria Achut Anib, Mayen Machut Achiek, Fabian Ndenzako, Olushayo Oluseun Olu

**Affiliations:** 1 Ministry of Health, Juba, South Sudan; 2 World Health Organization, Juba, South Sudan

**Keywords:** Sustainable development goal 3, universal health coverage, healthcare delivery, health system recovery, people-centred primary healthcare, South Sudan

## Abstract

Amidst the myriad of challenges that constrain good quality health care services delivery in the World’s youngest nation, South Sudan, there is a beacon of hope. The country’s revitalized peace agreement offers a new impetus for rebuilding the country, including its health system. Key achievements in the health care sector of the country such as development and implementation of a health sector strategic and health sector stabilization and recovery plans and implementation of a Boma Health Initiative programme which aims to scale up health services delivery at the community level provide a foundation on which acceleration of universal health coverage could rest. Other key achievements include polio-free certification of the country, significant reductions in the prevalence of Guinea Worm and other neglected tropical diseases and timely detection and response to the ongoing COVID-19 outbreak. Moving forward, attainment of universal health coverage in the country requires a strong and people-centred primary healthcare approach which will ensure that services reach the last mile. Bridging the humanitarian-development nexus is required to ensure accelerated recovery of the country’s health system. Furthermore, scaling up of community-based health initiatives such as the Boma Health Initiative as platforms for taking good quality health services to the hard-to-reach areas is imperative. This Journal Supplement highlights the key achievements and challenges on the road to universal health coverage in South Sudan and provides evidence-based information for rapidly scaling up health services provision.

## Editorial

Universal Health Coverage (UHC), a flagship target of the third Sustainable Development Goal (SDG3), is a situation where all persons have access to the required quality health services as at when and where they need them without having to encounter any financial barrier in doing so [1]. Three key elements are paramount for the attainment of UHC, namely availability of essential health services, including health promotion, curative and preventive services, good quality of and financial access to the health services [1]. South Sudan, the youngest nation globally, does not meet any of the three key elements of UHC. Inheriting a rudimentary health system at independence in 2011, the country has struggled to provide access to good quality health services to its people [2]. This is due to a major dearth of health care workers, non-functional supply chain management system for essential medicines and medical supplies, weak health coordination and oversight system which limits access to basic healthcare services [3]. Furthermore, the less than 3% of its GDP which is allocated to the health sector and out of pocket spending on health of over 50% is inadequate to fund healthcare services delivery in the country [4]. Thus, most of the available health care services are primarily provided by national and international non-governmental organizations (NGOs) which are largely funded externally [5].

The civil conflicts of 2013/14 and 2016 added further stress to an already fragmented health system resulting in further decimation of the healthcare system and erosion of the progress made in the first two years of independence and further limiting the country´s capacity to deliver good quality services [5]. The country´s public health indicators remain one of the lowest globally. As of 2017, access to healthcare services was 28% [2]. Immunization coverage is less than 50%, while life expectancy at birth, maternal mortality ratio and under-five mortality are respectively 58 years, 789 per 100,000 live births and 92.6 per 1000 live births [6,7]. Other factors outside the health system such as weak transport infrastructure, climatic conditions and insecurity due to recurrent inter-communal clashes that render more than half the country physically inaccessible by land for several months of the year further compound the country´s inability to achieve UHC.

However, there is a ray of hope in the country´s health sector even in the face of this grim situation. The revitalized peace agreement has brought a new impetus for rebuilding the country, including its health system. A health sector strategic plan (HSSP) has been developed and is being implemented, albeit slowly. In 2019, health partners supported the Ministry of Health in developing and implementing a health sector stabilization and recovery plan that aims to strengthen the health system resilience through accelerated implementation of the HSSP. A Boma Health Initiative (BHI) programme which aims to scale up health services delivery at the community level has been launched with support from partners [8]. The production, absorption and retention of various cadres of human resources for health are being accelerated. In addition, several strategic documents such as the health financing strategy, basic package of health and nutrition services and national health accounts have been developed and implemented to support the attainment of UHC and health-related SDGs [9].

Following an intensive childhood immunization programme using routine and supplementary strategies, the country and four other African countries were certified wild poliovirus free in 2020 [10]. Significant reductions have also been achieved in the prevalence of Guinea Worm and other neglected tropical diseases [11]. Several public health surveys which generated baseline data for evidence-based planning, implementation, supervision, monitoring and evaluation of essential health services have been conducted [12]. A national action plan for health security, including an effective disease surveillance system that provides timely detection and response to all disease outbreaks, has also been developed and implemented [13]. This enabled the country to prevent cross border transmission of the Ebola virus disease (EVD) in the Democratic Republic of Congo [14] and timely detect and respond to the Corona Virus Disease (2019) (COVID-19) outbreak in April 2020 [15].

**Way forward: strategies to fast-track South Sudan´s journey towards UHC:** moving forward, the country needs to sustain these modest achievements and use them as opportunities to build the foundation for UHC attainment for its people. This requires a strong and people-centred primary health care approach that will ensure that services reach the last mile. Several strategies are required in this regard. First, the humanitarian-development nexus should be bridged by ensuring that ongoing humanitarian response lay a foundation for longer-term health system strengthening and resilience building and vice versa. Second, using a well-coordinated approach to provide systematic stabilization and recovery of the country´s health system is required. Third, the scale-up of community-based health initiatives such as the BHI as platforms for taking health services to the last mile is imperative. Fourth, using innovative, appropriate and sustainable technologies such as digital health to accelerate health services delivery would be critical in expanding health services to hard-to-reach areas. Fifth, increased advocacy for political commitment to increase domestic funding and development of stronger partnerships among all stakeholders, especially the international organizations are required to increase the financing of the health sector. Lastly, strengthened capacity for health stewardship, coordination and evidence-based strategic planning and efficient allocation and use of resources is critical.

The public health articles in this journal supplement provide evidence for rapid implementation of the foregoing recommendations. The successes, challenges and recommendations for advancing the public health agenda in the country in thematic areas such as health emergency preparedness and response, immunization including polio eradication, prevention and control of neglected tropical diseases are presented and discussed. For instance, the lessons learnt from the successful implementation of essential health programmes such as the polio eradication initiative which are presented could be used to guide stabilization and recovery of the country´s health system towards achieving UHC.

**Ethical clearance and approval:** administrative clearance for publication of this editorial was provided by the Ministry of Health of South Sudan with Research Ethics Review Board (MOH-RERB) number MOH/RERB/D.03/2022. Ethical clearance was provided by WHO (WHO e-Pub no: ePub-IP-00331600-EC).
